# Definition of the Minimal Contents for the Molecular Simulation of the Yeast Cytoplasm

**DOI:** 10.3389/fmolb.2019.00097

**Published:** 2019-10-02

**Authors:** Vijay Phanindra Srikanth Kompella, Ian Stansfield, Maria Carmen Romano, Ricardo L. Mancera

**Affiliations:** ^1^School of Pharmacy and Biomedical Sciences, Curtin Health Innovation Research Institute, Curtin Institute for Computation, Curtin University, Perth, WA, Australia; ^2^Physics Department, Institute for Complex Systems and Mathematical Biology, University of Aberdeen, Aberdeen, United Kingdom; ^3^Institute of Medical Sciences, University of Aberdeen, Aberdeen, United Kingdom

**Keywords:** macromolecular crowding, proteomics, protein translation, yeast, molecular dynamics

## Abstract

The cytoplasm is a densely packed environment filled with macromolecules with hindered diffusion. Molecular simulation of the diffusion of biomolecules under such macromolecular crowding conditions requires the definition of a simulation cell with a cytoplasmic-like composition. This has been previously done for prokaryote cells (*E. coli*) but not for eukaryote cells such as yeast as a model organism. Yeast proteomics datasets vary widely in terms of cell growth conditions, the technique used to determine protein composition, the reported relative abundance of proteins, and the units in which abundances are reported. We determined that the gene ontology profiles of the most abundant proteins across these datasets are similar, but their abundances vary greatly. To overcome this problem, we chose five mass spectrometry proteomics datasets that fulfilled the following criteria: high internal consistency, consistency with published experimental data, and freedom from GFP-tagging artifacts. Using these datasets, the contents of a simulation cell containing a single 80S ribosome were defined, such that the macromolecular density and the mass ratio of ribosomal-to-cytoplasmic proteins were consistent with experiment and chosen datasets. Finally, multiple tRNAs were added, consistent with their experimentally-determined number in the yeast cell. The resulting composition can be readily used in molecular simulations representative of yeast cytoplasmic macromolecular crowding conditions to characterize a variety of phenomena, such as protein diffusion, protein-protein interactions and biological processes such as protein translation.

## Introduction

The environment inside cells is densely packed, termed macromolecular crowding, the extent of which varies throughout the different growth and differentiation stages of the cell, as well as according to its type and volume (Nakano et al., 2014). A typical cell has a macromolecular concentration in the range 100–450 g/L, with 5–40% of its volume being occupied by macromolecules (Feig et al., 2017). Therefore, the space available for the free diffusion of metabolites and other macromolecules is greatly reduced, leading to what is known as an excluded volume effect. This reduces diffusion and favors more compact protein conformations and protein association. Transient aggregation of proteins is favored in crowded systems and is correlated with slower diffusion (Nawrocki et al., 2017). Macromolecules reduce the amount of bulk-like water in the cell by reducing the amount of water molecules present beyond the second solvation layer (Harada et al., 2012). As a consequence, a 40% reduction in the dielectric constant of yeast cells compared to that of a dilute solution has been determined (Asami et al., 1976; Tanizaki et al., 2008), leading to an increase in electrostatic interactions between molecules. Hindered diffusion due to macromolecular crowding, on the other hand, increases the probability of ligands being in the vicinity of their receptors in what is termed caging effect, which enhances reaction rates (Feig et al., 2017). Cells are believed to maintain their macromolecular concentration within a very small range in a process now termed “homeocrowding” (Van Den Berg et al., 2017). Moreover, it has been shown that the diffusion coefficient of molecules depends not only on the macromolecular concentration but also on the composition of the solution (Wang et al., 2010). Molecular crowding inside cells affects various biochemical processes such as protein translation. The diffusion of tRNA complexes in the cytoplasmic environment is hindered by crowding, in turn affecting the rate of translation (Klumpp et al., 2013).

Molecular dynamics (MD) simulations can be used to characterize the complex nature of the effects of macromolecular crowding, including effects on the diffusion of tRNAs and their binding to cytoplasmic ribosomes during translation. Two prior studies of the cytoplasm have focused on prokaryotic systems (*E. coli*). In one study, 118 protein molecules were chosen on the basis of their mole percentage in the cytosol, with the number of ribosomes being scaled down based on abundances reported at cell level and a total macromolecular density of 340 g/L (Ridgway et al., 2008). Each protein molecule was represented as a sphere, whilst tRNAs were not included at all (Ridgway et al., 2008). In a second study, 51 different types of macromolecules were considered, out of which 45 were proteins and which accounted for 86% of the total cytoplasmic protein mass reported by the proteomics dataset used with a macromolecular concentration of 275 g/L. The simulation cell also included three types of tRNAs (tRNA-Gln, tRNA-Phe, and tRNA-Cys) and 10 ribosomes in their corresponding subunits. The volume corresponding to lipids, lipopolysaccharides, mRNA, DNA, murein, and glycogen was accounted for by increasing the concentration of protein in the simulation cell (McGuffee and Elcock, 2010). In a more recent cytoplasmic model, developed for *Mycoplasma genitalium*, the simulation cell comprised more than 1,000 protein molecules, 275 tRNAs, nucleotides, metabolites, ions, and a total of 26 million water molecules represented atomistically with a macromolecular density of 291.5 g/L (Feig et al., 2015). To our knowledge, an equivalent representative definition of the eukaryotic cytoplasm has not been reported in the literature. The key challenges in defining such a simulation cell include identification of the required proteomics datasets and defining appropriate criteria to minimize the size of the cell whilst retaining the properties of the cytoplasmic environment.

In this study, we sought to address the lack of a standard molecular simulation environment for eukaryotes by defining the contents of a simulation cell based on the abundances of proteins, tRNAs and ribosomes in the yeast cytoplasm. A recent yeast proteomics dataset (Ho et al., 2018) unified abundance data from 21 different datasets, comprising a range of mass spectrometry (MS)-derived datasets, datasets based on green fluorescent protein (GFP)-tagging of yeast proteins and GFP flow cytometry and also a tandem affinity purification (TAP-tagging)-immunoblot dataset. We employed an in-depth proteomics survey of these datasets in order to define a molecular simulation environment for a model eukaryote cell. However, these datasets vary in terms of the growth conditions used to culture the cells, the cellular growth phase, the units in which abundances are reported, and the technique used to measure them. It was therefore necessary to investigate how these factors affect protein abundances reported across the range of datasets. We characterized the internal consistency amongst the datasets and their agreement with other published experimental data, leading to the selection of a proteome composition for the yeast cytoplasmic environment. Consideration of additional experimental data on the macromolecular density and the mass ratio of ribosomal-to-cytoplasmic proteins in the cytoplasm was also used, allowing the definition of the contents of a molecular simulation cell representative of the yeast cytoplasm.

## Methods

### Definition of a Eukaryote Cell Simulation Environment

Previous reports of the number of ribosomes in yeast cytoplasm were taken from cell population scale experiments (Waldron and Lacroute, 1975) and from cell tomography experiments at single cell level (Yamaguchi et al., 2011), and were compared with the numbers calculated from proteomics datasets. The volume percentage of individual components of the yeast cell were also obtained from cell tomography studies (Yamaguchi et al., 2011), which are in agreement with other cell tomography experiments (Wei et al., 2012). Furthermore, we used the recently published unified yeast proteomics dataset that covers a total of 5,391 proteins (Ho et al., 2018).

Proteins associated with the nucleus, cell wall, ribosomes, mitochondria, endoplasmic reticulum, and vacuoles were removed from the dataset with the help of GO-slim annotations (http://www.yeastgenome.org/) to assign cellular location to a given protein. Gene ontology analysis of the function of encoded proteins was performed using the webserver Funcassociate 3.0 (http://llama.mshri.on.ca/funcassociate/) (Berriz et al., 2009).

### Statistical Analysis

The abundances reported for individual ribosomal proteins by any dataset were treated as multiple observations of the number of ribosomes (described in detail in the Results section). Based on this, pairwise statistical two-tailed *t*-tests for unequal variances between proteomics datasets were performed using an in-house code in MATLAB (https://github.com/BMMG-Curtin/FMOLB) to quantitatively understand the differences and similarities between datasets (Figure S1). Where multiple pairwise *t*-tests were conducted, the Bonferroni correction was applied to address type-I errors, whereby the critical alpha value is divided by the number of pairwise tests. In addition, *p*-values were adjusted using the Benjamini-Hochberg approach to address type-I errors and the results obtained were found to be qualitatively the same (Figure S1). The data was assumed to be normally distributed whilst conducting the above *t*-tests; therefore, a non-parametric Mann–Whitney *U*-test with the Bonferroni correction was also employed (Figure S2). The results of the *U*-test were also found to be qualitatively similar to the results obtained with the *t*-tests. Pairwise correlations between the functional ontological classes of proteins across different datasets were quantified using the Pearson's correlation coefficient. The Jaccard index was used to quantify the similarities between the ontological profiles obtained for each of the datasets.

## Results

### Analysis of Internal Consistency of Yeast Proteomics Datasets

In order to define the protein composition of a eukaryote molecular simulation cell, the recently published unified yeast proteomics dataset was used (Ho et al., 2018). This covers 5,391 genes with a total protein mass per yeast cell of 2.7 × 10^12^ Da, which is in good agreement with the total protein mass of a yeast cell previously reported to be 3 × 10^12^ Da (Sasidharan et al., 2012). This proteomics dataset comprises data integrated from 21 different datasets, which vary in the type of growth medium used to culture cells, their growth phase and the technique used to measure protein abundances.

**Figure 1:**
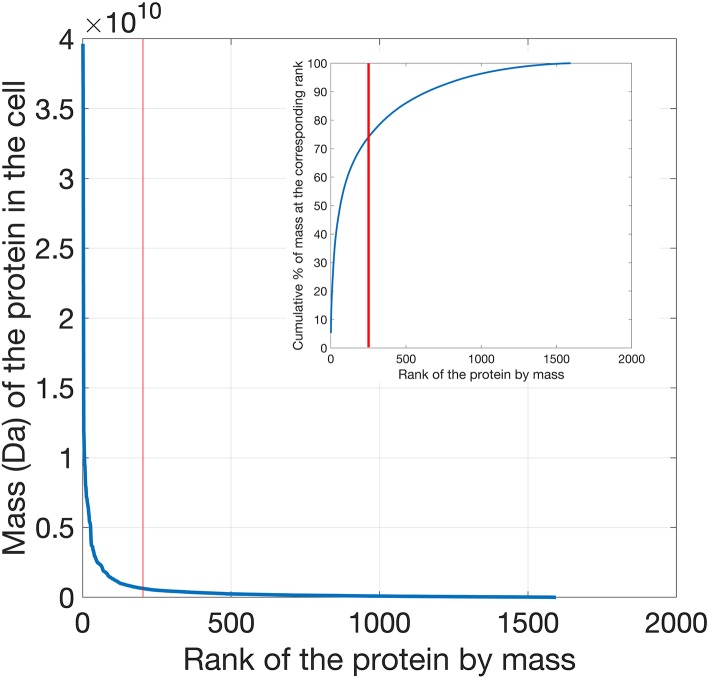
Distribution of protein mass (calculated as the product of molecular weight times abundance) per cell plotted as a function of the mass rank of each protein. Proteins in the yeast proteomics dataset were ranked according to their mass, exhibiting a clear exponential decrease as a function of their mass rank in the cell. In the inset the cumulative percentage of mass is plotted as a function of rank. The top 200 cytoplasmic proteins contribute to ~70% of the total cell protein mass.

**Figure 2:**
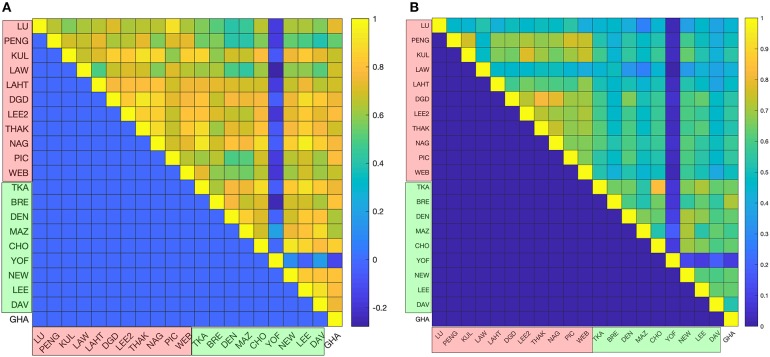
Statistical analyses of proteomics datasets. **(A)** Pairwise correlations between the ontological profiles obtained for the individual datasets. Correlations were measured using the Pearson correlation coefficient, whose values are color-coded (from the highest correlation in yellow to the lowest correlation in blue). **(B)** The ontology profile overlap between datasets is quantified using the Jaccard index and the color-code is the same as in the previous panel. In both panels mass spectrometry based datasets are indicated in red on the axes labeled as LU (Lu et al., 2007), PENG (Peng et al., 2012), KUL (Kulak et al., 2014), LAW (Lawless et al., 2016), LAHT (Lahtvee et al., 2017), DGD (De Godoy et al., 2008), PIC (Picotti et al., 2013), LEE2 (Lee et al., 2011), THAK (Thakur et al., 2011), NAG (Nagaraj et al., 2012), and WEB (Webb et al., 2013); GFP datasets are shown in green on the axes and are labeled as TKA (Tkach et al., 2012), BRE (Breker et al., 2013), DEN (Denervaud et al., 2013), MAZ (Mazumder et al., 2013), CHO (Chong et al., 2015), YOF (Yofe et al., 2016), NEW (Newman et al., 2006), LEE (Lee et al., 2007), and DAV (Davidson et al., 2011); and the TAP-immunoblot dataset is shown in white on the axes and is labeled as GHA (Ghaemmaghami et al., 2003). The top 200 proteins are shown to have a similar gene ontology profile across all of the datasets.

The top 200 most abundant proteins were taken from each of the 21 datasets based on their mass (i.e., molecular mass multiplied by their abundance) and were found to account for ~70% of the total cytoplasmic protein mass (Figure 1). In order to assess the possible influence of cell culture conditions, growth phase and the method used to measure protein abundance on the composition of the yeast cytoplasm, the ontological classes of these proteins were assessed. The systematic names of these proteins were submitted to the Funcassociate 3.0 webserver, which detects over-representation of gene ontologies in a gene list. The number of proteins associated with each gene ontology class was identified for every dataset. Each pair of datasets was then compared by calculating the Pearson's correlation coefficient between the number of proteins associated with each gene ontology class. The Jaccard index was used to quantify the similarities between the sets of gene ontology classes obtained for every dataset. Despite the above differences between the datasets, a similar ontological landscape for the top 200 proteins in each of the datasets was observed, except for one dataset that used N-terminal GFP tagging, YOF (Yofe et al., 2016; Figure 2).

Although the gene ontology profiles of the top 200 cytoplasmic proteins are similar across datasets, significant differences in protein abundances were observed. For example, the average coefficient of variation (CV) (measured across the 21 datasets) for the cytoplasmic proteins is 78%. The differences are more marked in the case of ribosomal proteins (CV = 106%).

In order to investigate the internal consistency of the proteomic datasets and their agreement with other published data, ribosomal proteins were examined separately. The protein composition of ribosomes can be assumed to be fixed (Perry, 2007) and there are 79 ribosomal proteins per ribosome. Since the stoichiometry for each ribosomal protein with respect to the ribosome (Warner, 1999) is 1:1, it should be expected that the numbers of each of these ribosomal proteins in a given dataset will lie within a very small range. The identity of the ribosomal proteins was taken from the crystal structure of the eukaryotic ribosome (PDB code 4V88) (Ben-Shem et al., 2011). The CV of these proteins was computed in every dataset and the average CV of all MS datasets is 69%, whereas the average CV of GFP datasets is 103%, indicating better internal consistency in MS datasets compared to GFP datasets.

**Figure 3:**
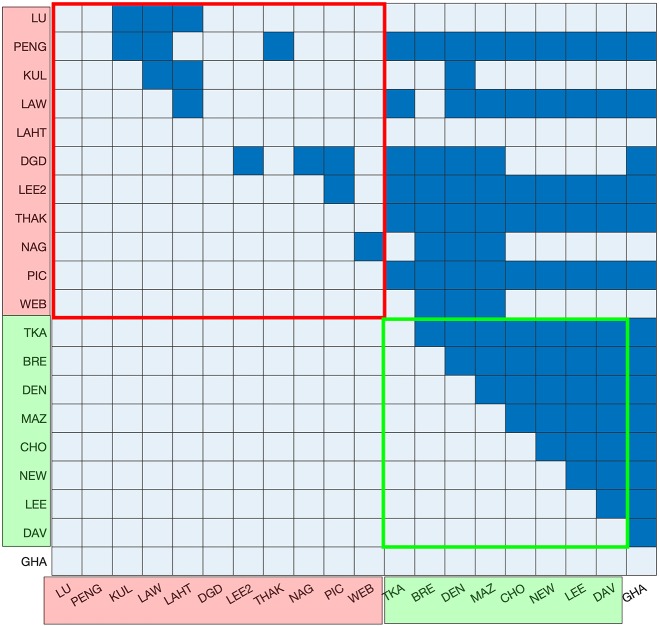
Testing of statistical difference between the abundance of ribosomal proteins in each of the datasets. Mass spectrometry-based datasets are shown in red on the axes, GFP datasets are shown in green on the axes and the TAP-immunoblot dataset is shown in white. Ribosomal protein numbers were not reported in the YOF dataset and, therefore, it is not included. The results of *t*-tests with *p* > (0.05/190) are colored dark blue and all others are colored light blue. GFP datasets exhibit a high level of consistency. There is also consistency among the first five MS datasets. However, there are no discernible patterns in terms of the growth media, growth phase or protein abundance units.

Depending on the consistency between datasets, the numbers reported for a given ribosomal protein across different datasets are expected to vary showing patterns in terms of experimental conditions. In order to test this, the abundances of different ribosomal proteins were compared across different datasets. Given the 1:1 stoichiometry for each ribosomal protein with respect to the ribosome (Warner, 1999), the abundance of each ribosomal protein in each dataset provided an estimate of the number of ribosomes per cell. The average number of ribosomal proteins was therefore calculated to derive an average ribosome per cell value for each dataset. The resulting values were then compared between datasets by performing multiple pairwise *t*-tests to determine any patterns arising from the growth media, growth phase or the technique used to measure protein abundance (Figure 3). High *p*-values were observed in the pairwise tests between the datasets derived from GFP-tagging of proteins, indicating consistency between them. On the other hand, no clear consistency was apparent within the MS datasets, and no patterns were observed that might be accounted for by the growth media or growth phase used during cell culture.

It has previously been reported that there are ribosomal proteins with extra-ribosomal functions in yeast (Lu et al., 2015). In order to test if the differences in the abundance (Table S1) of ribosomal proteins arise from the fact that some of them perform additional functions and might therefore be produced in excess of the requirements for ribosome synthesis, the mean of means and the mean of medians (across 21 datasets) of ribosomal proteins with extra functions (set I) and other ribosomal proteins (set II) were computed. If excess production of some ribosomal proteins was due to additional functions, their numbers might be expected to be higher than those of other proteins. However, the mean of means of set I is ~88,400 units, whilst that of set II is ~86,000 units. By contrast, the mean of medians of set I is ~61,700 and that of set II is ~53,157 units. Whilst ribosomal proteins with other functions seem to be abundant, it should be noted that the standard deviations of both sets of proteins are ~25,000. A *t*-test carried out comparing the means reported for ribosomal proteins in set I and set II has a *p*-value of 0.85 and a similar calculation with medians showed a *p*-value of 0.23. These high *p*-values suggest that the differences in mean/median abundances do not have statistical significance, suggesting that the differences in the abundances of ribosomal proteins are not due to the extra-ribosomal functions carried out by some of them. The causal relationships of this phenomenon will need to be further investigated.

### Selection of Datasets

Whilst the gene ontology profiles of the proteomics datasets are similar, they vary widely in the protein abundances reported. The ratio of the median of abundances reported by GFP datasets to the median of MS datasets was calculated for cytoplasmic and ribosomal proteins. We determined that for 74% of cytoplasmic proteins and 84% of ribosomal proteins the medians differ by more than 25%. The differences in the individual protein abundances between the GFP and MS datasets were reported to be possibly due to changes in protein or mRNA stability following GFP tagging (Ho et al., 2018). More specifically, in the case of ribosomal proteins, GFP tagging can alter their packing in the ribosome, thereby affecting their turnover dynamics and therefore their abundances (von der Haar, 2008).

The number of ribosomes, calculated by taking the median of all ribosomal proteins reported in the GFP datasets, revealed an estimated 51,800 ribosomes per cell, whereas previously reported figures are 150,000–300,000 (Waldron and Lacroute, 1975) and 169,000–265,000 (Yamaguchi et al., 2011) ribosomes per cell. As discussed earlier, the abundances of ribosomal proteins reported in the GFP datasets are also widely spread, with an average CV of 103%, in contrast to the average CV of 69% in the MS datasets. It was thus decided to omit the GFP datasets from further consideration.

The first five (LU, PENG, KUL, LAW, and LAHT) MS datasets report abundances in absolute numbers, whereas the other MS datasets report normalized abundances (with respect to the average of the five MS datasets) (Ho et al., 2018). When the median of the first five MS datasets was compared to the median of the other MS datasets individually for every protein, 78% of cytoplasmic proteins and 96% of ribosomal proteins showed more than 25% difference. These differences may potentially be an artifact of the normalization process. The number of ribosomes inferred from the median abundance of ribosomal proteins of the first five MS datasets was ~130,000, whereas it was only 30,500 when calculated from the other MS datasets. This latter, lower figure is significantly different to previous reports (Waldron and Lacroute, 1975; Yamaguchi et al., 2011), as discussed above. The five MS datasets also showed high internal consistency in the pairwise *t*-tests performed on ribosomal protein abundance compared to the other MS datasets (Figure 3). The five MS datasets were originally reported to be highly correlated (with the Pearson correlation coefficient varying from 0.43 to 0.81) (Ho et al., 2018), which is consistent with our findings. Consequently, it was decided that only the first five MS datasets would be used for the definition of the contents of a molecular simulation cell.

### Constraints for the Definition of the Contents of a Simulation Cell

A molecular simulation cell should be designed to mimic the environment of the yeast cytoplasm. This requires the inclusion of three important constraints: macromolecular density, the mass ratio of ribosomal-to-cytoplasmic proteins, and the number of ribosomes in the simulation cell.

Macromolecular density is an indirect measure of the excluded volume and, therefore, crowding. The volume of yeast cell has been reported to be 42 μm^3^ (Jorgensen et al., 2002) and from the cell tomography determinations (Yamaguchi et al., 2011) we estimated the cytoplasm in yeast to be 65% of the total cell volume (27.3 μm^3^). The mass of all the 1,374 cytoplasmic proteins in the dataset, excluding ribosomes, was calculated using the mean abundances of all proteins with the above chosen five MS datasets. There are 3 million tRNAs in a yeast cell (Waldron and Lacroute, 1975) and, using an average mass of 25,500 Da per tRNA (calculated assuming that there are 75 nucleotides in tRNAs, each weighing an average mass of 340 Da), the total tRNA mass was calculated. The median number of all ribosomal proteins across the five MS datasets was determined to be 126,213, which was used to calculate the ribosomal mass in the yeast cell. The total masses of tRNAs, ribosomes and cytoplasmic proteins was then used to estimate the macromolecular density of the yeast cytoplasm as 90 g/L.

It has been reported that the fractions of ribosomal protein (R-protein), translation protein (T-protein), fixed protein (Q), the proportion of which is independent of growth rate, and metabolic protein (P-protein), given by, Φ_R_, Φ_T_, Φ_Q_, and Φ_P_, respectively, are unique for a specific growth rate (Klumpp et al., 2013). Therefore,

(1) $$\begin{array}{l} \Phi_{\text{Q}}+\Phi_{\text{P}}=\frac{\text{Q }-\text{ Protein}}{\text{A}}+\frac{\text{P }-\text{ Protein}}{\text{A}}=C\left(growth\;rate\right) \end{array}$$

where A is the total protein mass and C is the growth rate specific constant. The total Q- and P-protein content can be divided into cytoplasmic and non-cytoplasmic fractions. Therefore, the previous equation can be rewritten as

(2) $$\begin{array}{l} \Phi_{\text{Q}}+\Phi_{\text{P}}=\frac{\text{non}-{\text{cytoplasmic}}_{\left(\text{Q}+\text{P}\right)}}{\text{A}}+\frac{{\text{cytoplasmic}}_{\left(\text{Q}+\text{P}\right)}}{\text{A}}\\ \;=C(growth\;rate) \end{array}$$

(3) $$\begin{array}{l} \frac{{\text{non }-\text{ cytoplasmic}}_{\left(\text{Q}+\text{P}\right)}}{\text{A}}:\;\frac{\text{cytoplasmi}{\text{c}}_{\left(\text{Q}+\text{P}\right)}}{\text{A}}=k\left(growth\;rate\right) \end{array}$$

The last Equation (3) states the assumption that the mass ratio of cytoplasmic to non-cytoplasmic proteins is constant at a given growth rate, from which it follows that cytoplasmic fraction in Q- and P-proteins remains constant. Since the T-protein fraction is a growth rate-dependent constant, the mass ratio of ribosomal-to-total cytoplasmic proteins is constant at a given growth rate. This is the second constraint for the definition of the contents of a simulation cell. The mass ratio of ribosomal-to-cytoplasmic proteins (rib/cyt) was determined to be 0.2229.

The crystal structure of the ribosome is composed of 75 ribosomal proteins (Ben-Shem et al., 2011) and, at such size, it would be computationally challenging to include multiple ribosomes in a single simulation cell. Equally, ignoring the contribution of the ribosome to the excluded volume and macromolecular density would affect the accuracy of a simulation. Therefore, addition of a single ribosome to the simulation cell was decided as the third constraint for the definition of its contents.

### Definition of the Contents of the Simulation Cell

The choice of five MS datasets reduced the number of cytoplasmic proteins with abundance data from 1,594 to 1,374; however, when calculating the macromolecular density of the cytoplasm, data from all 1,594 proteins was considered. The total mass of cytoplasmic proteins calculated using abundances in the unified dataset is 7.56 × 10^11^ Da. The median of the number of molecules reported for a given protein by the five chosen MS datasets was taken as the measure of its abundance in a typical yeast cell. The total mass of a given type of protein was calculated by multiplying its abundance (number of proteins per cell) by its molecular mass, and the protein list was then sorted in descending order of total mass. The top 200 proteins contribute, as mentioned earlier, about 70% of the total cytoplasmic protein mass. The top proteins from the list were chosen due to their significant contribution to the protein mass in the cytoplasm and their abundances were subsequently scaled down to their corresponding value in proportion to only one ribosome (calculated as the abundance “n” of a protein divided by the 126,213 ribosomes predicted in the MS datasets).

Each of the less abundant cytoplasmic proteins does not contribute significantly to the overall protein mass. However, their collective removal results in a significant loss in protein mass which needs to be accounted for in order to maintain the desired macromolecular density of the simulation cell. Additionally, a number of proteins will contribute to the cytoplasm in fractional units that are lost due to rounding. The number of protein molecules of each of the cytoplasmic proteins was thus multiplied by a scaling factor aimed at maintaining the overall macromolecular density of the simulation cell. The number of protein types was chosen such that their total mass contribution reflects the expected value of the rib/cyt ratio. This was achieved by testing multiple scaling factors under the above-described constraints. Use of a large scaling factor (e.g., 3.0) meant that the rib/cyt ratio could be reached with just 20 different types of proteins, amounting to 119 protein molecules. By contrast, the rib/cyt ratio could not be reached with very low scaling factors (e.g., <1.8). Although the total number of protein molecules remained in the range 120–130 with all of the scaling factors tested, the observed protein composition was affected significantly with the use of large scaling factors. A range of scaling factors meet the constraints of macromolecular density, rib/cyt ratio and the presence of one ribosome in the simulation cell. However, in order to maintain the most representative composition of cytoplasmic proteins, the lowest possible scaling factor of 1.803 was chosen. This resulted in a final list containing 128 protein molecules belonging to 70 types of proteins (Table S2).

Based on the constraint that there should be only one ribosome, the size of the simulation cell was calculated. A total of 126,213 ribosomes are assumed to be present in the cytoplasm, which has a volume of 27.3 μm^3^. This volume was scaled down to one ribosome unit, which for a cubic simulation cell results in a length of 560 Å. The number of tRNAs was scaled down from 3 million units per cell to the volume of the simulation box, resulting in 22 tRNA units. With one 80S ribosome, 128 protein molecules and 22 tRNAs, the resulting simulation cell has the required total macromolecular density of 90 g/L.

## Discussion

This study shows that the ontological profiles of the most abundant proteins in yeast remains constant despite differences in growth medium and growth phase, indicating that the most abundant proteins constitute the fundamental biochemical framework of the cell. The abundances reported in GFP datasets are affected by tagging, particularly in the case of ribosomal proteins. This has been explained previously on the basis that ribosomal proteins form a compact structure in a single ribosome molecule and the tag attached to them affects their packing. Although this explains the low numbers of ribosomal proteins reported, the cause of the high CV of ribosomal proteins in GFP datasets (CV = 103%), indicating a selective effect of tagging, compared with that of MS datasets (CV = 69%) remains unclear. Moreover, the average number of ribosomes calculated using MS datasets that report abundances in relative units is very low (30,500 units). The causes behind this remain undetermined, although normalization of the data is a possible factor.

Unlike prokaryotic cells, eukaryotic cells have a sophisticated organization of cellular machinery into different organelles with varying macromolecular environments. In order to study the influence of this macromolecular environment, an accurate description of its composition is needed. This was achieved by assigning the cellular location of a protein from its gene annotation data (GO-slim data) and determining the volume percentage of cytoplasm in yeast from cell tomography experiments. The macromolecular density of yeast cytoplasm was found to be 90 g/L, which is three times lower than that of the cytoplasm of *E. coli*. Measurements of the diffusion coefficient of GFP in eukaryotic and prokaryotic cells indicate that the eukaryotic cytoplasm is less crowded (Ellis, 2001), in line with our findings. Crowding in eukaryotic cells is also non-uniform. For example, in the nucleus we have calculated the protein density to be 346 g/L [using the 10–11 volume percentage obtained from cell tomography experiments (Yamaguchi et al., 2011) and nuclear protein abundances from the dataset (Ho et al., 2018)]. These large macromolecular density differences indicate that an accurate estimate of the macromolecular density of the organelle of interest is necessary.

In conclusion, a simulation cell was defined such that the yeast cellular composition of proteins, the ribosome-to-cytoplasmic protein mass ratio and the macromolecular density are retained. This was achieved by increasing the relative proportion of the most abundant proteins under specific constraints. The resulting simulation cell contains 128 protein molecules belonging to 70 protein types, 22 tRNAs and one 80s ribosome within a cubic cell of 560 Å in length. The simulation cell contents act as a generic representation of the cytoplasm that can be used to study the diffusion and interactions of molecules in the yeast cytoplasmic environment.

## Data Availability Statement

The datasets generated for this study can be found in the https://github.com/BMMG-Curtin/FMOLB.

## Author Contributions

VK conducted all of the analyses. VK and RM wrote the manuscript, which was further proofread by MR and IS. All authors conceived and designed this study and performed the interpretation of data.

### Conflict of Interest

The authors declare that the research was conducted in the absence of any commercial or financial relationships that could be construed as a potential conflict of interest.
