# Supplementary material for: Definition of the Minimal Contents for the Molecular Simulation of the Yeast Cytoplasm
Source: Front Mol Biosci. 2019 Oct 2;6:97. doi: 10.3389/fmolb.2019.00097 (PMC6783697; doi:10.3389/fmolb.2019.00097)
Supplement: Supplementary file 1 [file Data_Sheet_1.pdf]

Supplementary Information

Table S1. Mean abundances (averaged over 21 datasets) of ribosomal proteins.

Ribosomal protein	Mean abundance
RPL8A	188027.73
RPP0	130202.95
RPS7A	202553.26
RPS2	152569.09
RPL3	89802.65
RPL5	96389.57
RPS0A	115555.92
RPL4A	81725.33
RPS5	123613.53
RPS4A	103019.95
RPS3	102188.04
RPS1A	88136.51
RPS26A	181591.82
RPL22A	165100.58
RPL7A	80549.23
RPS18A	130570.40
RPL9A	102286.11
RPL10	85701.46
RPL19A	99641.26
RPL31A	162099.18
RPS8A	92966.31
RPS13	118213.17
RPS6A	72525.95
RPL36A	168796.39
RPS9A	81059.75
RPS20	119954.71
RPL18A	79537.21
RPS17A	103341.74
RPL25	103276.00
RPS11A	89415.05
RPS15	94003.66
RPL15A	61326.79
RPS12	92317.14
RPL28	83111.26
RPL2A	50517.13

RPL24A	74903.49
RPL17A	62827.69
RPL14A	80322.78
RPS16A	71760.17
RPL13A	50041.38
RPL21A	61140.15
RPS10A	82701.84
RPL35A	74545.27
RPL20A	50252.89
RPL43A	101502.12
RPS24A	65998.10
RPL11A	51004.52
RPS31	58390.66
RPS14A	68873.54
RPS25A	82158.97
RPL32	66342.15
RPL26A	66121.02
RPS19A	58515.32
RPL27A	59677.32
RPL33A	75015.30
RPL6A	44425.88
RPL30	74292.39
RPL39	126124.28
RPL16A	35233.79
RPS22A	53336.31
RPS21A	80034.68
RPL34A	55699.59
RPS23A	45213.45
RPL38	70481.00
RPL29	80524.73
RPS27A	58361.28
RPL42A	37748.62
RPS28A	57906.56
RPL23A	30148.74
RPL37A	42091.50
RPL41A	95917.02
RPS30A	38135.39
RPL40A	10587.84
RPS29A	18070.34
ASC1	155688.35
STM1*	78257.70

*STM1 is the non-ribosomal protein found in the crystal structure of ribosome.

Table S2. The final list of proteins and their structural information. The first column is the rank of the protein when the list is sorted in descending order of mass contributed to the simulation cell. Rows are colour-coded such that green denotes proteins that have an experimentally-determined structure (completely or partially), white denotes proteins that do not have structures but the structures can be predicted using homology modelling, and yellow denotes proteins that do not show sequence similarity to any known structure. There are structures readily available for 34 of the protein types, whilst 32 of the protein types show significant sequence identity with protein structures available and, therefore, their structures can readily be obtained using homology modelling. The remaining 4 types of proteins show no sequence similarity to any structures publicly available and, therefore, *ab initio* modelling approaches can be used to predict their structures.

	Systematic name	Standard name	Description	Number of molecules in simulation cell	PDB ID of the structure / template
1	YBR118W	TEF2	Translational elongation factor EF-1 alpha	13	1F60_A
2	YNL209W	SSB2	Cytoplasmic ATPase that is a ribosome-associated molecular chaperone	5	3GL1_A
3	YDR385W#	EFT2	Elongation factor 2 (EF-2), also encoded by EFT1	3	1N0U_A
4	YOL086C	ADH1	Alcohol dehydrogenase	8	4W6Z_A
5	YAL038W	CDC19	Pyruvate kinase	5	1A3W_A
6	YOR133W#	EFT1	Elongation factor 2 (EF-2), also encoded by EFT2	3	1N0U_A
7	YLR303W	MET15	O-acetyl homoserine-O-acetyl serine sulfhydrylase	5	2CTZ_A
8	YLR249W	YEF3	Translation elongation factor 3	2	2IWH_A
9	YER091C	MET6	Cobalamin-independent methionine synthase	1	3PPC_A
10	YKR059W	TIF1	Translation initiation factor eIF4A	3	2VSO_A
11	YLR109W	AHP1	Thiol-specific peroxiredoxin	6	4H86_A
12	YMR116C	ASC1	G-protein beta subunit and guanine dissociation inhibitor for Gpa2p	3	3RFG_A

13	YPL106C	SSE1	ATPase component of heat shock protein Hsp90 chaperone complex	1	3C7N_A
14	YAL003W	EFB1	Translation elongation factor 1 beta	4	1IJE_B
15	YPR074C	TKL1	Transketolase	1	1GPU_A
16	YLL039C	UBI4	Ubiquitin	2	4NNJ_B
17	YCL018W	LEU2	Beta-isopropylmalate dehydrogenase (IMDH)	2	3U1H
18	YER043C	SAH1	S-adenosyl-L-homocysteine hydrolase	2	1B3R
19	YDL229W	SSB1	Cytoplasmic ATPase that is a ribosome-associated molecular chaperone	1	3GL1_A
20	YJR109C	CPA2	Large subunit of carbamoyl phosphate synthetase	1	5DOT_A
21	YGL009C	LEU1	Isopropylmalate isomerase	1	4NQY
22	YML028W	TSA1	Thioredoxin peroxidase	3	3SBC_A
23	YDL055C	PSA1	GDP-mannose pyrophosphorylase	2	1TZF_A
24	YPL240C	HSP82	Hsp90 chaperone	1	2CG9_A
25	YLR058C	SHM2	Cytosolic serine hydroxymethyl transferase	1	5Z0Y_A
26	YJL138C	TIF2	Translation initiation factor eIF4A	2	1FUU_A
27	YDR502C	SAM2	S-adenosylmethionine synthetase	1	1O90_A
28	YDR023W	SES1	Cytosolic seryl-tRNA synthetase	1	3QNE_A
29	YHR064C	SSZ1	Hsp70 protein that interacts with Zuo1p	1	5MB9_A
30	YLL050C	COF1	Cofilin	3	1CFY_A
31	YPR145W	ASN1	Asparagine synthetase	1	1CT9_A
32	YCL030C	HIS4	Multifunctional enzyme containing phosphoribosyl-ATP pyrophosphatase	1	5VLB_A
33	YLR180W	SAM1	S-adenosylmethionine synthetase	1	2OBV_A
34	YOR027W	STI1	Hsp90 cochaperone	1	3UQ3_A
35	YHR019C	DED81	Cytosolic asparaginyl-tRNA synthetase	1	5XIX_A

36	YBR025C	OLA1	P-loop ATPase with similarity to human OLA1 and bacterial Ych	1	1NI3_A
37	YMR120C	ADE17	Enzyme of 'de novo' purine biosynthesis	1	1THZ_A
38	YBR126C	TPS1	Synthase subunit of trehalose-6-P synthase/phosphatase complex	1	5HUT_A
39	YGR124W	ASN2	Asparagine synthetase	1	1CT9_A
40	YLR027C	AAT2	Cytosolic aspartate aminotransferase involved in nitrogen metabolism	1	1YAA_A
41	YNL220W	ADE12	Adenylosuccinate synthase	1	5I33_A
42	YHR193C	EGD2	Alpha subunit of the nascent polypeptide-associated complex (NAC)	2	3MCE_A
43	YLR432W	IMD3	Inosine monophosphate dehydrogenase	1	5MCP_A
44	YMR217W	GUA1	GMP synthase	1	5TW7_A
45	YNL138W	SRV2	CAP (cyclase-associated protein	1	1K4Z_A
46	YBR143C	SUP45	Polypeptide release factor (eRF1) in translation termination	1	4CRN_X
47	YLR150W	STM1	Protein required for optimal translation under nutrient stress	1	
48	YKL035W	UGP1	UDP-glucose pyrophosphorylase (UGPase)	1	2I5K_A
49	YLR359W	ADE13	Adenylosuccinate lyase	1	5VKW_A
50	YOL058W	ARG1	Arginosuccinate synthetase	1	1VL2_A
51	YNL064C	YDJ1	Type I HSP40 co-chaperone	1	1NLT
52	YOR184W	SER1	3-phosphoserine aminotransferase	1	6CZY_A
53	YGL105W	ARC1	Protein that binds tRNA and methionyl- and glutamyl-tRNA synthetases	1	4R1J_A

54	YPL037C	EGD1	Subunit beta1 of the nascent polypeptide-associated complex (NAC)	1	NO
55	YKL142W	MRP8	Protein of unknown function; undergoes sumoylation; transcription induced under cell wall stress	1	
56	YDL192W	ARF1	ADP-ribosylation factor	1	5A1U_A
57	YER055C	HIS1	ATP phosphoribosyl transferase	1	2VD3_A
58	YIL041W	GVP36	BAR domain protein	1	
59	YFL045C	SEC53	Phosphomannomutase	1	5UE7_A
60	YEL021W	URA3	Orotidine-5'-phosphate (OMP) decarboxylase	1	3GDK_A
61	YDL137W	ARF2	ADP-ribosylation factor	1	1MR3_F
62	YDR533C	HSP31	Methylglyoxalase that converts methylglyoxal to D-lactate	1	4QYX_A
63	YBR109C	CMD1	Calmodulin	1	6OQQ_B
64	YLR172C	DPH5	Methyltransferase required for synthesis of diphthamide	1	3I4T_A
65	YNL079C	TPM1	Major isoform of tropomyosin	1	
66	YDR071C	PAA1	Polyamine acetyltransferase	1	1B6B_A
67	YGL106W	MLC1	Essential light chain for Myo1p	1	1M45_A
68	YDR177W	UBC1	Ubiquitin-conjugating enzyme	1	1TTE_A
69	YIL138C	TPM2	Minor isoform of tropomyosin	1	5ND5_A
70	YPL225W		may interact with ribosomes, based on co-purification experiments	1	2JYN_A
71	YMR260C	TIF11	Translation initiation factor eIF1A	1	3J80_i

*STM1 is added as a component of ribosome.
#These proteins are paralogs encoded by different genes.

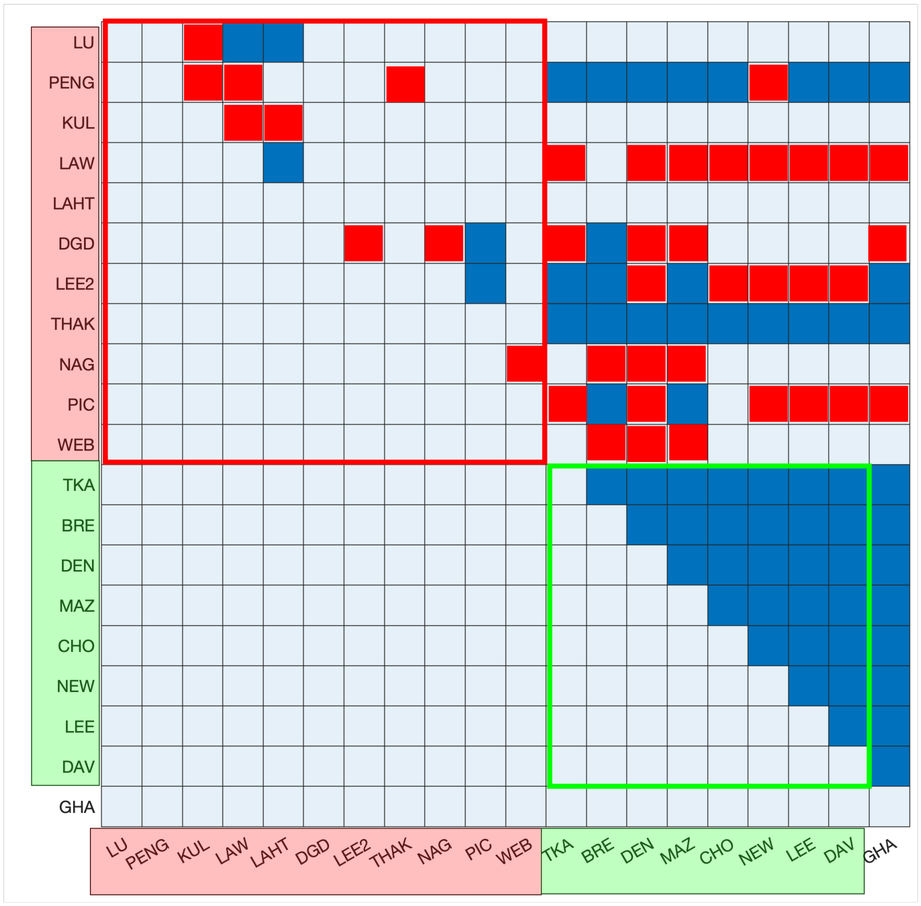

Figure S1. Results of the t-tests corrected for type-I errors using the Benjamini-Hochberg approach (an alternative to the Bonferroni correction). Dataset pairs for which p-values > 0.05 are coloured in dark blue. Squares in red show deviations from the t-test predictions. The results are qualitatively similar to the t-test predictions and the conclusions drawn from t-tests remain valid.

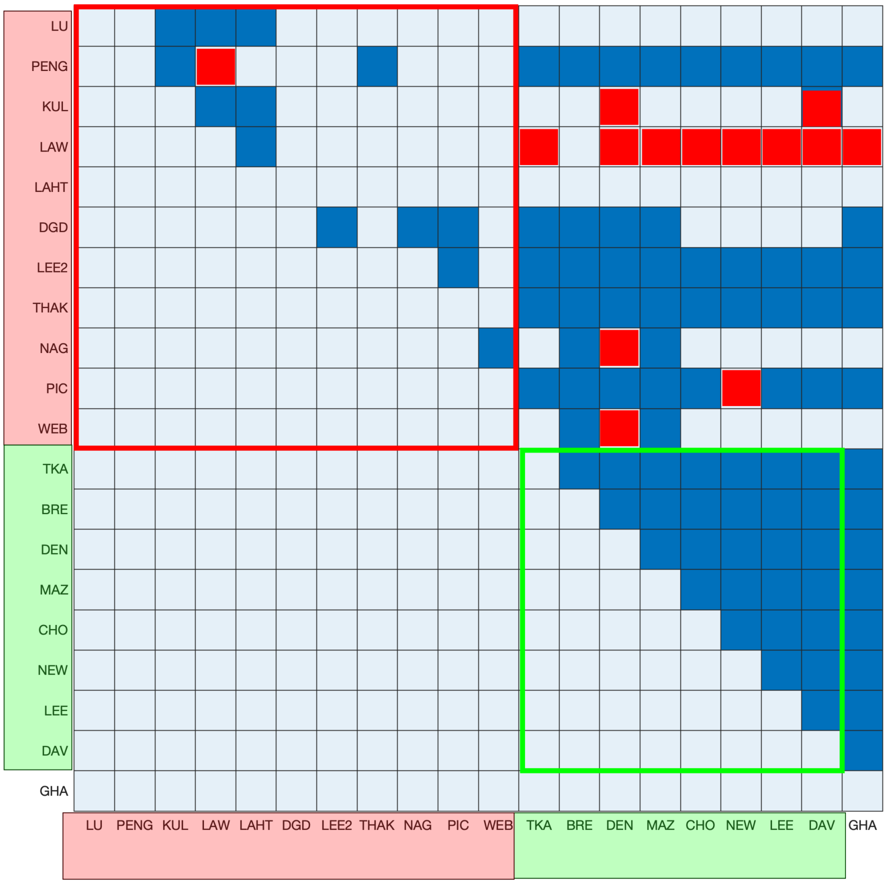

Figure S2. Results of the Mann-Whitney *U* test performed in pairwise manner across the datasets. The Bonferroni correction was applied to address type-I errors. Squares in dark blue show p-values > (0.05/190). Squares in red show the dataset pairs for which the p-values predicted using the Mann-Whitney *U* test are different from the p-values predicted with the t-tests. The results are qualitatively similar to the t-test results and the conclusions drawn from the t-tests remain valid.
